# Under-treatment of elderly patients with ovarian cancer: a population based study

**DOI:** 10.1186/s12885-015-1947-9

**Published:** 2015-11-26

**Authors:** Elisabeth Fourcadier, Brigitte Trétarre, Claudine Gras-Aygon, Fiona Ecarnot, Jean-Pierre Daurès, Faïza Bessaoud

**Affiliations:** 1 Cancer Registry of Hérault Departement of France - ICM, 208, rue des apothicaires, 34298 Montpellier, Cedex 5 France; 2 Department of Cardiology, EA3920, University Hospital Besançon, Besançon, France

**Keywords:** Ovarian cancer, Elderly, Treatment pattern, Guidelines-recommended therapy, Survival

## Abstract

**Background:**

Ovarian cancer is the fourth most common cancer among women in France, and mainly affects the elderly. The primary objective of this study was to compare treatment of ovarian cancer according to age.

**Methods:**

All patients with invasive cancer (*n* = 1151) diagnosed between 1997 and 2011 in the Herault Department of southern France were included. Demographic data (age, area of residence), cancer characteristics (stage, histology, grade) and treatment modality (type, period and location of treatment) were analysed. Univariate and multivariate logistic regression was used to compare treatment by age.

**Results:**

Ovarian cancer was less treated in elderly compared to younger patients, regardless of the type of treatment. This difference was more pronounced for chemotherapy, and was maximal for surgery followed by chemotherapy (odds ratio (OR) for surgery for patients aged >70 vs those aged <70 years = 0.47 [0.24–0.91], OR for chemotherapy, age >70 vs <70 = 0.30 [0.16–0.55] and OR for surgery plus chemotherapy, age >70 vs <70 = 0.14 [0.08–0.28]). This effect of age was independent of other variables, including stage and grade. The probability of receiving standard treatment, in accordance with recommendations, was reduced by 50 % in elderly patients compared to their younger counterparts. Overall and net survival of elderly patients with standard treatment was similar to those of younger patients treated outside standard treatment.

**Conclusions:**

Elderly women with ovarian cancer were therapeutically disadvantaged compared to younger women. Further studies including co morbidities are necessary to refine these results and to improve therapeutic management of elderly patients with ovarian cancer.

## Background

Cancer is the second leading cause of death in women after cardiovascular disease. In France, the number of new cancer cases increased significantly between 1980 and 2012 [1]. This increase is largely due to the increase in population and aging, which automatically increases the number of cases, particularly those occurring in the elderly. In Western countries, ovarian cancer remains the leading gynecological cause of death [2]. Ovarian cancer represents the 6^th^ cause of cancer and the 4^th^ cause of death in women in France. Nearly 4600 new cases are diagnosed, and nearly 3100 women die of this disease annually in France [1].

Ovarian cancer is a disease that is most common in the elderly, and its incidence increases with age to reach a peak during the 7^th^ decade of life [3]. The populations in western countries tend to be aging; hence, the number of elderly women needing to be treated for ovarian cancer can be expected to increase [4].

Prior studies have shown that elderly women, compared to their younger counterparts, are more likely to have a delayed diagnosis or an advanced stage at diagnosis [5–7]. Furthermore, in elderly patients, the tumor characteristics (such as histology and stage) are often not investigated [8]. Furthermore, treatment of this disease is complex, consisting in consensus-based treatment defined according to stage and tumor grade. Surgery and/or chemotherapy constitute the cornerstone of ovarian cancer treatment [9]. Such taxing treatment requires good physiological capacity, and many older people may have diminished physiological reserves, especially due to the presence of comorbidities. Consequently, many authors have addressed the issue of treatment of ovarian cancer in older women, highlighting significant disparities in senior health care. Several studies [4, 10–12] have shown that elderly patients with ovarian cancer have a poorer prognosis compared to their younger counterparts, due in part to under-treatment. Furthermore, when the elderly are treated, standard treatment guidelines are less frequently applied than for younger patients, and the elderly receive less aggressive therapy, even in the absence of comorbidity [13–15].

Based on these findings, our study hypothesis was that elderly women with ovarian cancer are under-treated. The main objective of this work was to investigate whether treatment practices differed between elderly women with ovarian cancer and their younger counterparts.

## Methods

### Study population

This observational study included all ovarian cancer cases (ICD-O-3 code C56.9) (*n* = 1151) occurring among women diagnosed between January 1997 and December 2011, residing in the Hérault department of southern France. We excluded all patients with borderline tumors.

### Data recorded

The tumor registry has existed in the Hérault department since 1983 and covers a population of more than one million inhabitants (1 062 036 according to the 2011 census). The registry records all cases of cancer occurring in this department in a continuous and exhaustive manner. All cases of ovarian cancer included in the present study were validated through systematic verification across original medical records. Demographic data, tumor characteristics (date of incidence, stage and grade at diagnosis) and treatment data (actual treatment given and location of treatment), as well as survival were determined in a reliable manner. This study was approved by two Ethics Committees for studies using human subjects (the Consultative Committee on Data Processing for Medical Research and the National authority for the protection of privacy and personal data) which provided approval to access routinely collected population-based cancer data in Hérault and which advocates that all medical information is confidential and anonymous and the need for individual informed consent was waived on the basis that the registry collects data already available in the patients’ medical files.

### Variables

#### Outcome variable

The primary outcome variable analyzed in this study was the type of treatment. Firstly, this variable was used in binary form, i.e. patient had surgery (yes/no) and patient had chemotherapy (yes/no). Subsequently, it was analyzed in the form of treatment sequences or patterns. Four patterns were used (surgery alone, surgery followed by chemotherapy, chemotherapy alone or neo adjuvant, and no treatment). These patterns were derived from treatment guidelines and recommendations corresponding to the existing consensus for the treatment of ovarian cancer. The treatment strategy depended on the tumour stage and grade. Surgery was generally the first line treatment, with a view to full tumour resection. Surgery alone may be sufficient to treat early grade cancers (stages IA/IB and grade 1). For intermediate stage cancers (stages IC, II, III and grades 1–3), surgery is followed by chemotherapy with a view to eliminating any residual cancer cells and reducing the risk of relapse. When the tumour is diagnosed at an advanced stage (stage IV), chemotherapy may be performed before surgery to reduce the size of the tumour before resection, or may be used as a stand-alone approach. Finally, in our last model, we created a binary variable to record whether standard treatment was applied (yes/no), according to whether guidelines-recommended treatment was applied or not.

#### Explanatory variables

The main explanatory variable in this study was the age at diagnosis, divided into two classes, namely ≤70 years and >70 years old. Other variables considered were demographic data and cancer characteristics (stage, grade, histology). We recorded the area of residence (urban or rural). Tumour stage was recorded and classified into four groups according to the TNM classification system and International Federation of Gynecology and Obstetrics (FiGO) staging system (UICC 6th edition). In our study, tumor histology was classified according the World Health Organization (WHO) classification and grouped into epithelial type; other; or “unknown histology”, when there was no microscopic confirmation or no histological testing.

The tumor grade was coded in three categories: well-differentiated (grade 1), moderately differentiated (grade 2), and poorly differentiated (grade 3) or unknown. The healthcare establishment where the first treatment took place was categorized into: private for-profit clinics, public university teaching center (Public Univ.), public non-academic (Public non Univ.) and Unknown or Outside the Hérault department (Unknown).

The study period was divided into three classes: before (≤2002), during (2003–2007) and after (≥2008) the national cancer plan. The national cancer plan mobilizes a national foundation for the organization healthcare and prevention, and provides support for research in the field of cancer. This plan includes the creation of an oncogeriatrics task force responsible for the promotion and coordination of projects in the field of epidemiology, prevention and adaptation of treatments and clinical trials in the elderly population [16]. The variables recorded for survival analysis included the date of diagnosis, the date of last contact and vital status (alive/deceased). Vital status was obtained from National Institute of Statistics and Economic Studies (INSEE) and the National Directory for the Identification of Individuals (RNIPP). The descriptive and comparative part of this study covers the period 1997–2011 and the survival analysis covers the period 1997–2010, with follow-up ending on 30/06/2013. Our descriptive and comparative analysis focuses on 1151 cases and the survival analysis on 1056 cases (692 deaths and 364 censored).

### Statistical analysis

The chi square or Fisher’s exact test were used, as appropriate, to compare cancer characteristics according to age. Univariate and multivariate analysis using logistic regression were used to identify the association between treatment (Surgery and Chemotherapy: yes/No) and age, adjusted for other factors such as stage, grade, histological research, treatment location, and area of residence. Multivariate logistic regression was used to analyze the treatment pattern (surgery alone, surgery followed by chemotherapy, chemotherapy alone or neo-adjuvant chemotherapy, *vs.* no treatment) according to age. The Kappa test was used to assess agreement between the treatment administered and the treatment recommended by current guidelines. Overall survival was estimated according the Kaplan-Meier method. Net survival (survival if the cause of death under consideration was the only cause of death) is now recommended as a substitute for relative survival methods in current use, to evaluate population-based cancer survival [17, 18]. We estimated net survival using the non-parametric Pohar Perme estimator [19], which is based on the mortality rate of the general population and provides unbiased estimates of net survival. Overall survival curves were compared using the log rank test. All tests were two-sided and a *p*-value <0.05 was considered statistically significant. A Cox regression model was used to estimate hazard ratios for overall mortality, with adjustment for age and other confounding factors including grade and stage. We verified the conditions of application of the model by testing the proportionality of risks. All statistical analyses were performed with the R software package. Net survival analysis was performed using the “relsurv” package specific to relative survival analysis.

## Results

Among 1151 women diagnosed with invasive ovarian cancer, 38.9 % were elderly (≥70 years old). The average age was 64.7 years, median age was 66 years.

Table 1 shows the characteristics of ovarian cancer according to age at diagnosis. Compared to younger women, elderly patients more frequently had their cancer diagnosed at a more advanced stage (*p* <0.0001); 12 % of elderly women presented stage I, compared to 24.5 % for younger women. Histological testing was often not performed or unknown in elderly women (*p* < 0.001) and the cancer grade was also more often unknown (*p* < 0.001). Younger women tended to be treated in Public university hospitals, whereas elderly patients tended to be treated in private clinics, although the difference was not statistically significant (*p* = 0.06). The period of diagnosis and the area of residence did not differ according to age.

**Table 1:** Characteristics of ovarian cancer patients according to age

Age	≤70 years (N = 703)		>70 years (N = 448)		*P* value for Chi2
	N	%	N	%	
Stage					<0.0001
I	172	24.5 %	54	12.1 %	
II	47	6.7 %	24	5.4 %	
III	328	46.7 %	227	50.7 %	
IV	122	17.4 %	115	25.7 %	
Unkown	34	4.8 %	28	6.3 %	
Grade					0.0003 (0.0676*)
1	62	8.8 %	19	4.3 %	
2	120	17.1 %	61	13.6 %	
3	150	21.3 %	79	17.6 %	
Unkown	371	52.8 %	289	64.5 %	
Histology					<0.0001
Epithelial	655	93.2 %	379	84.6 %	
Other	39	5.6 %	8	1.8 %	
No histology	9	1.3 %	61	13.6 %	
Treatment location					0.0137 (0.0676*)
Private	386	54.9 %	265	59.1 %	
Public Univ.	240	34.1 %	120	26.8 %	
Public non-Univ.	51	7.3 %	33	7.4 %	
Unkown	26	3.7 %	30	6.7 %	
Period of diagnosis					0.5509
≤2002	279	39.7 %	164	36.6 %	
2003–2007	220	31.3 %	144	32.1 %	
≥2008	204	29,0 %	140	31.3 %	
Area of residence					0.5343
Urban	598	85.6 %	387	86.4 %	
Rural	105	14.9 %	61	13.6 %	

*p-value when modality ‘Unknown’ was excluded from analysis

Tables 2 and 3 show the associations between treatment (surgery or chemotherapy) and age, and other potential confounding factors. Compared to their younger counterparts, elderly women less often underwent surgical treatment (60.9 % versus 89.6 %, *p* < 0.001) and chemotherapy (57.4 % versus 76.4 %, *p* < 0.001). After adjustment for cancer characteristics (stage, grade, histology and other adjuvant treatments), we observed that elderly women were 3.6 less likely to have surgical treatment (OR = 0.28 [0.19–0.41]) and three times less likely to undergo chemotherapy (OR = 0.34 [0.24–0.48]).

**Table 2:** Univariate and multivariate analysis of the association between demographic and cancer characteristics and treatment by surgery

		Surgery (N = 903)		No surgery (N = 243)		*p*	Univariate analysis	Multivariate analysis
		N	%	N	%		OR (95%CI)	OR^a^ (95%CI)
Age	≤70	630	89.6 %	73	10.4 %	<0.0001	1	1
	>70	273	60.9 %	175	39.1 %		0.18 (0.13–0.25)	0.28 (0.19–0.41)
Stage								
	I-II	295	99.3 %	2	0.7 %	<0.0001	125.59 (30.5–516.35)	153.42 (35.67–659.9)
	III	448	80.7 %	107	19.3 %		3.56 (2.56–4.97)	4.46 (2.97–6.72)
	IV	128	54.0 %	109	46.0 %		1	1
	Unkown	32	51.6 %	30	48.4 %		0.91 (0.52–1.6)	3.69 (1.33–10.25)
Adjuvant treatment	yes	677	84.3 %	126	15.7 %	<0.0001	2.94 (2.17–3.85)	2.51 (1.58–3.97)
	no	226	64.9 %	122	31.5 %		1	1
Histology						<0.0001		
	Epithelial	856	82.8 %	178	17.2 %		1	1
	Other	46	97.9 %	1	2.1 %		9.56 (1.31–69.81)	8.4 (1.05–67.44)
	No histology	1	1.4 %	69	98.6 %		0.003 (0.001–0.02)	0.01 (0.14–0.79)
Grade						<0.0001		
	1	73	90.1 %	8	9.9 %	(0.4595*)	1	1
	2	763	97.7 %	18	2.3 %		0.99 (0.41–2.39)	1.94 (0.72–5.28)
	3	198	86.5 %	31	13.5 %		0.70 (0.31–1.59)	1.63 (0.63–4.19)
	Unkown	469	71.1 %	191	28.9 %		0.27 (0.13–0.57)	0.82 (0.35–1.96)
Treatment location								
	Private	543	83,4 %	108	16.6 %	0.0001	1.37 (0.99–1.89)	2.00 (1.30–3.08)
	Public Univ.	283	78,6 %	77	21.4 %	(0.0015*)	1	1
	Public non-Univ.	57	67,9 %	27	32.1 %		0.57 (0.34–0.97)	0.65 (0.31–1.34)
	Unkown	20	35,7 %	36	64.3 %		0.15 (0.08–0.28)	0.33 (0.14–0.79)
Period of diagnosis								
	≤2002	348	78.6 %	95	21.4 %	0.4751		
	2003–2007	292	80.2 %	72	19.8 %			
	≥2008	263	76.5 %	81	23.5 %			
Area of residence								
	Rural	132	79.5 %	34	20.5 %	0.7184		
	Urban	771	78.3 %	214	21.7 %			

*OR (95%CI)* Odd Ratio and 95 % Confidence Interval, *OR*^*a*^ Odd Ratios were adjusted for factors associated with treatment with a *p*-value <0.10 by univariate analysis

*p-value when modality ‘Unknown’ was excluded from analysis

**Table 3:** Univariate and multivariate analysis of the association between demographic and cancer characteristics and treatment by chemotherapy

		Chemotherapy (N = 794)		No chemotherapy (N = 357)		*p*	Univariate	Multivariate
		N	%	N	%		OR (95 % CI)	OR^a^ (95 % CI)
Age	≤70	537	76.4 %	166	23.6 %	<0.0001	1	1
	>70	257	57.4 %	191	42.6 %		0.42 (0.32–0.54)	0.34 (0.24–0.48)
Stage								
	I-II	147	49.5 %	150	50.5 %	<0.0001	0.36 (0.25–0.52)	0.13 (0.08–0.21)
	III	459	82.7 %	96	17.3 %		1.77 (1.23–2.54)	1.28 (0.83–1.97)
	IV	173	73.0 %	64	27.0 %		1	1
		15	24.2 %	47	75.8 %		0.12 (0.06–0.23)	0.14 (0.06–0.32)
Adjuvant treatment	yes	677	74.2 %	235	25.8 %		3.03 (2.22–4)	2.60 (1.67–4.05)
	no	117	49.0 %	122	51.0 %	<0.0001	1	1
Histology						<0.0001		
	Epithelial	765	74.0 %	269	26.0 %		1	1
	Other	17	36.2 %	30	63.8 %		0.20 (0.11–0.37)	0.37 (0.18–0.76)
	No histology	12	17.1 %	58	82.9 %		0.07 (0.04–0.14)	0.23 (0.11–0.48)
Grade						<0.0001		
	1	48	59.3 %	33	40.7 %		1	1
	2	144	79.6 %	37	20.4 %		2.68 (1.51–4.74)	2.01 (1.04–3.89)
	3	197	86.0 %	32	14.0 %		4.23 (2.37–7.56)	2.85 (1.48–5.50)
	Unkown	405	61.4 %	255	38.6 %		1.09 (0.68–1.75)	1.31 (0.75–2.27)
Treatment location								
	Private	467	71.7 %	184	28.3 %	<0.0001	0.91 (0.68–1.22)	1.15 (0.81–1.63)
	Public Univ.	265	73.6 %	95	26.4 %		1	1
	Public non-Univ.	40	47.6 %	44	52.4 %		0.33 (0.20–0.53)	0.41 (0.23–0.75)
	Unkown	22	39.3 %	34	60.7 %		0.23 (0.13–0.42)	0.37 (0.17–0.78)
Period of diagnosis								
	≤2002	298	67.3 %	145	32.7 %	0.1637	1	1
	2003–2007	265	73.0 %	98	27.0 %		1.30 (0.96–1.77)	1.78 (1.23–2.57)
	≥2008	231	67.2 %	113	32.8 %		0.99 (0.74–1.34)	1.26 (0.87–1.83)
Area of residence								
	Rural	115	69.3 %	51	30.7 %	0.9297		
	Urban	679	68.9 %	306	31.1 %			

*OR (95%CI)* Odd Ratio and 95 % Confidence Interval, *OR*^*a*^ Odd Ratios were adjusted for factors associated with treatment with a *p*-value <0.10 by univariate analysis

^*^p-value when modality ‘Unknown’ was excluded from analysis

Based on the assumption that the association between treatment and age could differ according the stage at diagnosis, we performed a stratified analysis, which showed that the association between age and surgery was significantly different according to cancer stage. For early stages I-II, surgical treatment did not differ according age (OR >70 vs ≤70 = 0.17 [0.01–4.45], *p* = 0.2858). However, for stage III, there was a significant association between surgery and age, with elderly patients having a significantly lower likelihood of surgery (OR >70 vs ≤70 = 0.17 [0.09–0.30]), and there was a similar, albeit non-significant trend for stage IV (OR >70 vs ≤70 = 0.56 [0.31–1.03]). The association between chemotherapy and age was also stratified according stage at diagnosis, but there were no significant differences. The regression models including an interactive term between age and stage confirmed the findings of our stratified analysis.

With the exception of the area of residence, all variables were significantly related to the three different treatment pathways (surgery alone, surgery + chemotherapy, chemotherapy alone or neo-adjuvant) (Table 4). Multivariate regression adjusted for cancer characteristics (stage, grade, histology), treatment location and period of diagnosis showed that there was a significant difference between elderly patients and their younger counterparts in terms of all treatment sequences. Regardless of the treatment pattern, elderly patients received less treatment. We observed a gradient effect between treatment and age linked to the introduction of chemotherapy and its association with surgery: for surgery OR >70 vs ≤70 years = 0.46 [0.16–0.55], for chemotherapy OR >70 vs ≤70 years = 0.29 [0.16–0.55] and OR >70 vs ≤70 years = 0.13 [0.08–0.25] for surgery plus chemotherapy (data not shown).

**Table 4:** Demographic and cancer characteristics by treatment patterns

		No treatment		Surgery alone		Surgery + Chemotherapy		Chemotherapy alone or neo adjuvant		*P*-value
	Mod.	N	%	N	%	N	%	N	%	
Age	<= 70 years	28	3.9 %	138	19.7	402	57.3	134	19	<0.0001
	>70 years	96	21.5 %	93	20.9 %	142	31.9	115	25.8	
Stage	1-II	1	0.3 %	149	50.2 %	139	46.8 %	8	2.7 %	<0.0001
	III	50	9.0 %	45	8.1 %	325	58.7 %	134	24.2 %	
	IV	46	19.6 %	17	7.2 %	70	29.8 %	102	43.4 %	
	Unkown	27	43.6 %	20	32.3 %	10	16.1 %	5	8.1 %	
Grade	1	2	2.5 %	31	38.3 %	34	41.9 %	14	17.3 %	<0.0001
	2	4	2.2 %	32	17.8 %	108	60 %	36	20 %	
	3	9	3.9 %	22	9.7 %	133	58.3 %	64	28.1 %	
	Unkown	109	16.5 %	146	22.2 %	269	40.8 %	135	20.5 %	
Histology	No histology	58	82.9 %	0	0 %	1	1.4 %	11	15.7 %	<0.0001
	Other	0	0 %	30	63.8 %	16	34.0 %	1	2.1 %	
	Epithelial	66	6.4 %	201	19.5 %	527	52.1 %	237	22.9 %	
Period of diagnosis	<=2002	46	10.4 %	99	22.4 %	219	49.6 %	78	17.7 %	0.0003
	2003–2007	34	9.4 %	64	17.7 %	193	53.2 %	72	19.8 %	
	> = 2008	44	12.9 %	68	19.8 %	132	34.5 %	99	28.9 %	
Treat.	Private	41	6 %	141	21.7 %	336	51.8 %	131	20.2 %	0.0001
location	Public Univ	33	9.2 %	62	17.	163	45.4 %	101	28.1 %	
	Public non-Univ	20	23.8 %	24	28.6 %	30	35.7 %	30	11.9 %	
	Unknown	30	53.6 %	4	7.1 %	15	26.8 %	7	12.5 %	
Area of residence	Rural	17	10.3 %	34	20.6 %	82	49.7 %	32	19.4 %	0.8609
	Urban	1077	10.9 %	197	20.0 %	462	47 %	217	22.1 %	

The concordance test (data not shown) showed that younger patients had a 34 % [26–42 %] chance of receiving guidelines-recommended therapy, whereas elderly patients had only a 19 % [11–27 %] chance, *p* <0.001. By univariate analysis, the variables associated with the application of standard (guidelines-recommended) treatment were age (*p* <0.0001), stage (*p* = 0.0019) and histology (*p* = 0.0443). Multivariate analysis showed that elderly patients had more than 50 % less likelihood of receiving guidelines-recommended therapy (OR = 0.48 [0.36–0.64]) (Table 5).

**Table 5:** Multivariate analysis of the factors associated with application of guidelines-recommended therapy

		Standard treatment applied = No		Standard treatment applied = yes		OR^a^ (95 % CI)
		N	%	N	%	
Age	<= 70	169	30.2 %	390	69.8 %	1
	>70	153	47.9 %	166	52,0 %	0.48 (0.36–0.64)
Stage	I-II	60	31.4 %	131	68.6 %	1
	III	172	34.6 %	325	65.4 %	1.81 (1.2–2.81)
	IV	90	47.4 %	100	52.6 %	1.64 (1.16–2.33)
Grade	1	24	32.8 %	49	67.1 %	1
	2	56	32.9 %	114	67.1 %	1.17 (0.64–2.14)
	3	73	34.1 %	141	65.9 %	1.17 (0.64–2.11)
	Unknown	169	40.1 %	252	59.9 %	0.95 (0.55–1.67)
Histology	No histology	6	60,0 %	4	40,0 %	1
	Epithelial	305	35.9 %	544	64.1 %	0.62 (0.17–2.3)
	Other	11	57.9 %	8	42.1 %	0.37 (0.14–0.98)
Treatment location	Private	188	35.9 %	335	64.1 %	1.33 (0.97–1.82)
	Public Univ.	122	39.7 %	170	60.3 %	1
	Public non-Univ.	16	32,0 %	34	68,0 %	1.56 (0.78–3.06)
	Unknown	6	26.1 %	17	73.9 %	0.96 (0.54–1.67)

*OR (95%CI)* Odd Ratio and 95 % Confidence Interval, *OR*^*a*^ Odd Ratios were adjusted for factors associated with treatment with a *p*-value <0.10 by univariate analysis

**Fig. 1:**
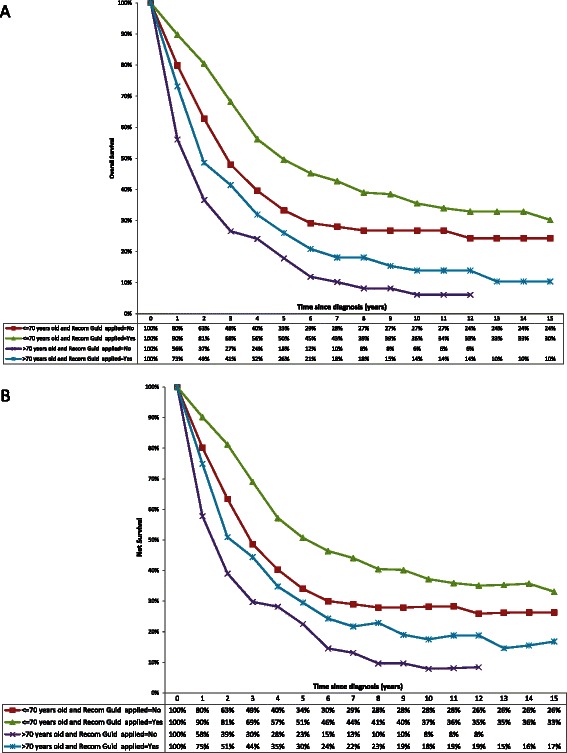
10-year overall survival (panel **a**) and net survival (panel **b**) by age and treatment recommendation

For the survival analysis, 1056 women were followed between 1997 and 2010, including 692 deaths and 364 censored. The median was 39.4 months for overall survival, and 39.9 for net survival. Overall and net survival plotted by the Kaplan Meier stratified according to the implementation (or not) of standard guidelines-recommended therapy, showed that elderly patients had poorer survival than younger patients (log-rank test *p* < 0.001), all the more so when guidelines-recommended was not administered in the elderly (Fig. 1a and b). Use of guidelines-recommended therapy improved survival in elderly women, bringing it up to a level almost equivalent to survival in younger women who did not receive guidelines-recommended treatment. There was no significant difference in use of guidelines-recommended therapy in older vs younger women for survival durations <10 years (*p* = 0.20). However, a significant difference was observed when overall survival exceeded 10 years (*p* = 0.02) and a trend towards a difference was observed for net survival (*p* = 0.08). In a Cox regression model for overall mortality, including terms for age, stage, grade, histology and standard of treatment (Table 6), older age (>70 years) was associated with an almost twofold increase in the risk of death, and stage III and stage IV disease were associated with significantly greater risks of death compared with stage I disease (hazard ratios of 3.8 and 5.2, respectively). Application of standard (guidelines-recommended) therapy was also associated with a significant reduction in the relative risk of death (hazard ratio = 0.74) after adjustment for age, stage, grade and histology.

**Table 6:** Adjusted effect of age on overall mortality by multivariate Cox regression analysis

		HR	95 % CI
Age	<= 70	1	
	>70	1.96	(1.65–2.33)
Stage	I–II	1	
	III	3.78	(2.84–5.03)
	IV	5.18	(3.77–7.12)
Grade	1	1	
	2	1.36	(0.90–2.05)
	3	1.05	(0.69–1.58)
	Unknown	1.38	(0.84–2.02)
Histology	No histology	1.52	(0.80–2.88)
	Epithelial	1	
	Other	1.35	(0.69–2.64)
Standard treatment	No	1	(0.62–0.89)
	Yes	0.74	

*HR (95%CI)* Hazard Ratio and 95 % Confidence Interval

## Discussion

Our study shows that elderly women with ovarian cancer receive less treatment than their younger counterparts, regardless of the type of treatment considered. We found that the difference between age groups was greater for chemotherapy than for surgery, and was highest for the combination of surgery plus chemotherapy. The cancer characteristics being equal, elderly women had less chance of receiving standard therapy, i.e. the recommended treatment by current guidelines. Elderly patients in whom guidelines-recommended treatment was not applied had poorer likelihood of survival as compared to elderly patients who received guidelines-recommended therapy, and as compared to younger women. In line with previous reports [4–6, 13, 20, 21], our findings also show that ovarian cancer in elderly women were more often discovered at an advanced stage as compared to younger women, and are less often submitted to histological testing.

We found that elderly women with ovarian cancer were three times less likely to have chemotherapy than younger women. As regards surgery, elderly patients were 3.6 times less likely to have surgical treatment than their younger counterparts. The association between surgical treatment and age was different according to cancer stage at diagnosis, since treatment differences were age-related for the higher stages rather than lower stages. Our results are in line with several previous studies [4, 5, 11, 13, 14, 22], and in particular, our findings are concordant with two recent studies [11, 13] showing that age is an independent predictor of non-administration of either surgery or chemotherapy.

Despite medical progress, and the introduction of a national cancer plan by the French government in 2003 to promote better management of cancer patients and highlight the problem of health inequity, our study, like several others [5, 11, 13, 14, 20], found that elderly patients were less likely to be treated as aggressively as younger patients. The association between treatment and age showed a gradient effect linked to the use of chemotherapy. Indeed, compared to younger patients, elderly women had less surgery, and the chances of being treated decreased even more when chemotherapy was the treatment of choice, and even further still when chemotherapy was combined with surgery. Furthermore, we showed than standard guidelines-recommended therapy was less frequently applied in the elderly, and hence, elderly patients were 50 % less like to receive standard therapy than younger patients. The reasons for this discrepancy could be related to more advanced disease, or to functional impairment in the elderly, which may be incompatible with implementation of recommended treatment. However, some studies have reported that age remains an independent predictor of under-treatment. Recent reports [4, 11, 14, 23] have suggested that elderly patients are much less likely to receive standard treatment, even after adjusting for comorbidities. Maas et al reported in a population of patients with FIGO II or III ovarian cancer that elderly (≥70 years) patients were seven times less likely to receive guidelines-recommended treatment (surgery + chemotherapy) than younger patients, even after adjustment for comorbidities (OR ≥70 vs <70 years = 0.14 [0.07 to 0, 21] and OR comorbidities vs no comorbidities = 0.91 [0.78 to 0.93]) [14]. This suggests that under treatment cannot be explained by poorer functional status in elderly patients. Other potential explanatory factors could include patient and/or physician choices.

The strengths of this study include its 14 year coverage of all cancer cases in the Herault Department, the relatively large size (1151 incident cases) and its population-based nature. We analyzed the variable “treatment” in the form of treatment sequences, which is rarely found in the literature, and confirmed our hypothesis that elderly women with ovarian cancer are under-treated. Another strongpoint is the use of a variable to take compliance with guidelines into account, by evaluating the application of guidelines-recommended therapy. This variable allowed us to highlight that older women were two times less likely to receive guidelines-recommended treatment than younger women, and this had a deleterious effect on overall survival. Indeed, overall survival in elderly women who did not receive standard treatment was worse than overall survival younger women, but it was also worse than elderly women who did receive standard treatment.

Our study suffers from some limitations. The analysis did not take into account factors such as comorbidities or functional status because these variables were not available in the registry. However, the fact that overall survival in this study was similar to the net survival (defined as the survival that would be observed if cancer was the only possible cause of death) suggests that the effect of comorbidities could be negligible, and probably did not modify the independent effect of age on treatment.

In our study, we chose to present the results in terms of net survival, as calculated using the Pohar Perme estimator [19]. This estimator of net survival has been shown in the literature to be more appropriate for registry data [17, 18]. This is due to the fact that classical “relative-survival” methods used to estimate net survival provide biased estimates. The Pohar-Perme net survival estimator may be prone to random variation and result in biased estimates when exact follow-up times are not available, or when follow-up is incomplete [24]. However, this situation is avoided in our study, since survival data (i.e. follow-up times) recorded in the registry was exhaustive, we have exact data on the number of deaths or patients lost to follow-up, with the exact dates when the events occurred. Our analysis is therefore not at risk of biased estimates, since the exact follow-up times are known, and the follow-up is fully complete.

Second, there was a considerable proportion of missing data. However, our data show that diagnostic tests (grade, stage, histology) are more often overlooked in elderly patients vs their younger counterparts. The fact that there are many patients with unknown histology or unknown grade is precisely the reflection of this phenomenon, as this indicates that the diagnosis is not pursued in detail to obtain this information in elderly patients. This could also be explained by the fact that the group of patients who underwent surgery comprises patients who had surgery after having chemotherapy, which may render analysis of the surgical specimens more difficult, or even impossible, thus leading to more missing data.

Furthermore, no information about the reasons for non-treatment, or the treatment modalities (e.g. type of surgery: complete or incomplete debulking) was available in our data. As highlighted by literature data [25], there exist large inequalities in access to healthcare and this is particularly valid for the elderly. We cannot exclude the fact that elderly patients differ with respect to their tolerance of treatment and their comorbidities, and some elderly patients might yield benefit from more aggressive therapy than they actually receive in reality. Previous studies [21, 26, 27] have indicated that in general, the elderly tolerate surgery well and are able to tolerate chemotherapy in the same conditions and modalities (schedule, dosage, complete courses…) as their younger counterparts. Therefore, age itself does not constitute the sole reason for withholding treatment. Although age was found to have an independent effect on under-treatment in our study, further research is warranted to evaluate whether this effect persists when individual comorbidities and treatment patterns (e.g. type of surgery, duration of treatment, completion of treatment,…) are accounted for, as these are all factors that may contribute to under-treatment of elderly patients.

Other variables that were not available in our study may also influence the association between treatment and age. One study reported that being cared for by a gynecologic oncologist influenced the type of treatment administered, and for advanced stages (FIGO III or IV), treatment by a gynecologic oncologist increased the probability of receiving chemotherapy twofold [10]. Other variables such as marital status or socio-economic status also deserve be taken into account, since some studies have suggested that single people or people with a low level of education are more likely to have chemotherapy [15].

## Conclusion

In conclusion, our study showed that elderly were under treated. For similar cancer characteristics, the probability of undergoing guidelines-recommended treatment was two-times lower in elderly patients compared to the younger counterparts. Elderly patients have improved likelihood of survival when recommended treatment is applied. Further research, however, is warranted to investigate whether the independent effect of age persists in the presence of co-morbidity. Acknowledging the under-treatment of elderly patients constitutes a first step in the broader process of improving therapeutic management of these patients. Several possibilities for improving patterns of care have been proposed. Geriatric assessment could help determine which patients are too frail to undergo standard treatment, and is essential for the initiation of treatment, particularly burdensome therapy.

## Abbreviations

OR: Odd Ratio
HR: Hazard Ratio
CI: Confidence Interval

## References

[1] Binder-Foucard F, Bossard N, Delafosse P, Belot A, Woronoff A-S, Remontet L (2014). Cancer incidence and mortality in France over the 1980-2012 period: solid tumors. Rev Dépidémiologie Santé Publique.

[2] Jemal A, Siegel R, Xu J, Ward E (2010). Cancer statistics, 2010. CA Cancer J Clin oct.

[3] Yancik R, Ries LG, Yates JW (1986). Ovarian cancer in the elderly: an analysis of Surveillance, Epidemiology, and End Results Program data. Am J Obstet Gynecol.

[4] Petignat P, Fioretta G, Verkooijen HM, Vlastos AT, Rapiti E, Bouchardy C (2004). Poorer survival of elderly patients with ovarian cancer: a population-based study. Surg Oncol.

[5] Ries LA (1993). Ovarian cancer. Survival and treatment differences by age. Cancer.

[6] Chi DS, Liao JB, Leon LF, Venkatraman ES, Hensley ML, Bhaskaran D (2001). Identification of prognostic factors in advanced epithelial ovarian carcinoma. Gynecol Oncol.

[7] Markman M, Lewis JL, Saigo P, Hakes T, Rubin S, Jones W (1993). Impact of age on survival of patients with ovarian cancer. Gynecol Oncol.

[8] de Rijke JM, Schouten LJ, Schouten HC, Jager JJ, Koppejan AG, van den Brandt PA (1996). Age-specific differences in the diagnostics and treatment of cancer patients aged 50 years and older in the province of Limburg, The Netherlands. Ann Oncol Off J Eur Soc Med Oncol ESMO.

[9] Aebi S, Castiglione M, ESMO Guidelines Working Group (2008). Epithelial ovarian carcinoma: ESMO clinical recommendations for diagnosis, treatment and follow-up. Ann Oncol Off J Eur Soc Med Oncol ESMO.

[10] Cress RD, O’Malley CD, Leiserowitz GS, Campleman SL (2003). Patterns of chemotherapy use for women with ovarian cancer: a population-based study. J Clin Oncol Off J Am Soc Clin Oncol.

[11] Jordan S, Steer C, DeFazio A, Quinn M, Obermair A, Friedlander M (2013). Patterns of chemotherapy treatment for women with invasive epithelial ovarian cancer--a population-based study. Gynecol Oncol.

[12] Chan JK, Urban R, Cheung MK, Osann K, Shin JY, Husain A (2006). Ovarian cancer in younger vs older women: a population-based analysis. Br J Cancer.

[13] Jørgensen TL, Teiblum S, Paludan M, Poulsen LØ, Jørgensen AYS, Bruun KH (2012). Significance of age and comorbidity on treatment modality, treatment adherence, and prognosis in elderly ovarian cancer patients. Gynecol Oncol.

[14] Maas HA, Kruitwagen RF, Lemmens VE, Goey SH, Janssen-Heijnen ML (2005). The influence of age and co-morbidity on treatment and prognosis of ovarian cancer: a population-based study. Gynecol Oncol.

[15] Carney ME, Lancaster JM, Ford C, Tsodikov A, Wiggins CL (2002). A population-based study of patterns of care for ovarian cancer: who is seen by a gynecologic oncologist and who is not?. Gynecol Oncol.

[16] Les Plans cancer de 2003 à 2013 - Plan cancer | Institut National Du Cancer [Internet]. [cité 28 sept 2015]. Disponible sur: http://www.e-cancer.fr/Plan-cancer/Les-Plans-cancer-de-2003-a-2013

[17] Danieli C, Remontet L, Bossard N, Roche L, Belot A (2012). Estimating net survival: the importance of allowing for informative censoring. Stat Med.

[18] Roche L, Danieli C, Belot A, Grosclaude P, Bouvier AM, Velten M (2013). Cancer net survival on registry data: use of the new unbiased Pohar-Perme estimator and magnitude of the bias with the classical methods. Int J Cancer.

[19] Perme MP, Stare J, Estève J (2012). On estimation in relative survival. Biometrics.

[20] Yancik R (1993). Ovarian cancer. Age contrasts in incidence, histology, disease stage at diagnosis, and mortality. Cancer.

[21] Hightower RD, Nguyen HN, Averette HE, Hoskins W, Harrison T, Steren A (1994). National survey of ovarian carcinoma. IV: Patterns of care and related survival for older patients. Cancer.

[22] Hershman D, Fleischauer AT, Jacobson JS, Grann VR, Sundararajan V, Neugut AI (2004). Patterns and outcomes of chemotherapy for elderly patients with stage II ovarian cancer: a population-based study. Gynecol Oncol.

[23] Uyar D, Frasure HE, Markman M, von Gruenigen VE (2005). Treatment patterns by decade of life in elderly women (> or =70 years of age) with ovarian cancer. Gynecol Oncol.

[24] Seppä K, Hakulinen T, Pokhrel A (2015). Choosing the net survival method for cancer survival estimation. Eur J Cancer Oxf Engl 1990.

[25] Bouchardy C, Rapiti E, Blagojevic S, Vlastos A-T, Vlastos G (2007). Older female cancer patients: importance, causes, and consequences of undertreatment. J Clin Oncol Off J Am Soc Clin Oncol.

[26] Bicher A, Sarosy G, Kohn E, Adamo DO, Davis P, Jacob J (1993). Age does not influence taxol dose intensity in recurrent carcinoma of the ovary. Cancer.

[27] Thigpen T, Brady MF, Omura GA, Creasman WT, McGuire WP, Hoskins WJ (1993). Age as a prognostic factor in ovarian carcinoma. The Gynecologic Oncology Group experience. Cancer.

